# Impact of bariatric surgery on the effectiveness of serological response after COVID-19 vaccination

**DOI:** 10.1007/s00423-022-02516-6

**Published:** 2022-04-29

**Authors:** Mohamed Hany, Bart Torensma, Anwar Ashraf Abouelnasr, Ahmed Zidan, Mohamed Ibrahim, Ann Samy Shafiq Agayby, Mohamed Hesham, Amel Elsheredy, Ghada Ahmed Abu-Sheasha

**Affiliations:** 1 Present Address: Department of Surgery, Medical Research Institute, Alexandria University, Alexandria, Egypt; 2 Present Address: Clinical Epidemiologist, Leiden University Medical Center (LUMC), Leiden, The Netherlands; 3 Department of Pharmacology, Alexandria University, Alexandria, Egypt; 4 Department of Microbiology, Medical Research Institute, Alexandria University, Alexandria, Egypt; 5 Department of Bio-Medical Informatics and Medical Statistics, Medical Research Institute, Alexandria University, Alexandria, Egypt

**Keywords:** Adverse events, Bariatric surgery, COVID-19, Obesity, Serology, Vaccination

## Abstract

**Purpose:**

The primary objective of the current study is to determine whether bariatric surgery reversed the negative impact of obesity on the serological response after the COVID-19 vaccination. This objective is achieved in two steps: (a) quantifying the negative impact of obesity on the serological response after COVID-19 vaccination if it is present, and (b) testing whether bariatric surgery reversed this impact. The secondary objective was to monitor the occurrence of adverse events.

**Methods:**

This is a prospective cohort study between May 2021 and August 2021 on the strength of serological response after COVID-19 vaccination. Patients were classified into three groups. Group A (controls with normal or overweight), Group B (bariatric patients pre-operative), and Group C (bariatric patients post-operative). Quantitative antibodies against SARS‑CoV‑2 RBD with a strong neutralizing capacity were quantified from sera after at least 2 weeks post-vaccination.

**Results:**

Of the 276 participants, Group A had n = 73, Group B had n = 126, and Group C had n = 77 patients. Overall, a strongly positive vaccine serological response was observed among 86% in group A, 63% in Group B, and 88% in Group C. Group C showed 5.33 times [95% CI 2.15 to 13.18] higher immune response than group B. Mild to moderate adverse events occurred in 30.1% [95% CI 24.7 to 35.9] of the study samples. Adverse events with the whole virus, mRNA, and vector vaccines occurred in 25%, 28%, and 37%, respectively.

**Conclusion:**

Vaccinating and bariatric surgery are safe and effective treatments in the serological response in patients who suffer from obesity.

## Introduction

The universal search for an effective vaccine began immediately when the World Health Organization (WHO) declared COVID-19 a global pandemic on March 11^th^, 2020 [1]. On December 11^th^, 2020, the US Food and Drug Administration (FDA) issued the first emergency use authorization for a vaccine for the prevention of COVID-19 [2, 3].

From January 2021, the Egyptian government supplied the AstraZeneca and Sinopharm vaccines to the public free of charge. As of 27^th^ of December 2021, 20.1% of the Egyptian population has been fully vaccinated with either vaccine [4]. In late 2021, the government also provided the Johnson & Johnson/Janssen vaccine to be administered upon request [5]. However, our bariatric center has also accommodated several patients from outside who have taken the Pfizer, Moderna, and Johnson and Johnson vaccines.

The effect of vaccines on the general population is well documented; however, there is insufficient data about their impact on this population. It is not yet known if the effect differs in patients with obesity or patients who have undergone bariatric surgery. Bariatric patients undergo a dramatic change in body composition, affecting vaccine efficacy throughout the patient’s journey. Patients with obesity are expected to have lower vaccine serological than underweight and normal-weight individuals [6]. Patients with obesity are also more prone to hospitalization, ICU admission to an intensive care unit, and mortality with COVID-19 than normal-weight patients [7], highlighting the urgent need for proper vaccination and weight management.

There has been much discussion on the ideal way to measure the vaccines’ effectiveness. However, vaccine serology is still measured by antibody levels in blood [8]. All vaccines aim to generate spike protein-specific antibodies, and all have been shown to induce anti-S IgG antibodies [9].

The primary objective of the current study was to determine whether bariatric surgery reverses the negative impact of obesity on the serological response after the COVID-19 vaccination. This objective was divided into two steps: (a) quantifying the negative impact of obesity on the serological response after COVID-19 vaccination if it is present, and (b) testing whether bariatric surgery reversed such an impact. The secondary objective was to monitor the occurrence of adverse events.

## Materials and methods

### Study design

This prospective cohort study was conducted at a single center between May 2021 and August 2021.

For analysis, study patients were divided into three study groups:

- Group A: Included healthcare workers who are healthy and not have obesity and therefore not indicated for bariatric surgery,
- Group B: Included patients who were prepared for bariatric surgery and still had not performed it yet.
- Group C: Included patients who had undergone a primary laparoscopic Roux-en-Y gastric bypass (RYGB) or sleeve gastrectomy surgery.

To explore whether bariatric surgery and weight loss improve the serological response, by comparing groups B and C (we hypothesized that Group C would have a stronger serological response than Group B). Group A was included to demonstrate the impact of obesity.

We found reports on the baseline serological response after COVID-19 vaccination among patients with obesity in Egypt. Thus, we conducted an interim power analysis after collecting a total sample size of 276 (Group A = 72, Group B = 126, and Group = 74). The immune response comprises three categories (negative or low positive, moderately positive, and high positive). The distribution of baseline serological response (in Group B) was 0.27, 0.10, and 0.63. With 80% power and an alpha of 0.05 using a two-sided test, the collected sample size was sufficient to detect a minimum (conservative) proportional odds ratio of 2.282. Therefore, we did not collect more data. This odds ratio value corresponds to a change of 16.5% in the percentage of patients who show a high positive serological response. (Patients in Group A and C powered together provide controls for Group B). Hmisc R package was used to calculate this power analysis [10].

### Data collection

Patients’ sociodemographic data, medical conditions, BMI, and history of infection were collected. The history of the previous infection was defined, according to the modified WHO surveillance case definition [11]: A person who has had the following symptoms within the last 10 days.

Acute onset of fever AND cough; OR Acute onset of any three or more of the following signs or symptoms: fever, cough, general weakness/fatigue, headache, myalgia, sore throat, coryza, dyspnea, anorexia/nausea/vomiting, diarrhea, and altered mental status. Furthermore, data on the type and time of surgery, type of vaccine, and timing of vaccination doses (single or complete dose) were collected. Additionally, blood samples were collected at least 2 weeks post-vaccination, as that was the approximate median time to seroconversion in previous reports [12, 13].

All patients provided written and oral informed consent. All data were used anonymously. The study was conducted according to the Declaration of Helsinki and approved by the Medical Research Institute’s ethical committee.

### Quantification of SARS‑CoV‑2 antibody response

Coronavirus genomes encode four main structural proteins: spike (S), envelope (E), membrane (M), and nucleocapsid (N). The S protein is a very large transmembrane protein that assembles into trimers to form the distinctive surface spikes of coronaviruses. The spike (S) protein plays the most important role in viral attachment, fusion, and entry through the binding of the S protein to the angiotensin-converting enzyme 2 (ACE2) and serves as a target for the development of antibodies, entry inhibitors, and vaccines. Each S monomer consists of an N-terminal S1 domain and a membrane-proximal S2 domain. S1 contains a receptor-binding domain (RBD) that can specifically bind to ACE2 receptors on target cells, and hence it typically represents the site of neutralizing antibodies quantification of anti-SARS-CoV-2 S protein RBD antibodies that can represent a useful tool to estimate the individual protection against SARS-CoV-2 infection [14]. According to the manufacturer’s instructions, Sera were separated and tested on the commercially available Elecsys® Anti-SARS-CoV-2 assay (Roche Diagnostics International Ltd, Rotkreuz, Switzerland) [15]. The Elecsys Anti-SARS-CoV-2 S is quantitative serologically that detects high-affinity antibodies to the SARS-CoV-2 S protein RBD and has a low risk of detecting weakly cross-reactive and non-specific antibodies. Quantitative antibodies against SARS‑CoV‑2 RBD with a strong neutralizing capacity were categorized into four categories as follows; negative (< 1 U/mL), low positive (1–5 U/mL), medium (> 5–10 U/mL), strong positive (> 10 U/mL) with a maximum cutoff value of 250 U/mL.

### Adverse events

Adverse events were graded according to the FDA’s guidance on adverse events for vaccines which grades clinical and laboratory abnormalities as mild (Grade 1), moderate (Grade 2), severe (Grade 3), or (Grade 4) [16].

### Statistical methods

We used both descriptive statistics and inferential statistics. All data were first tested for normality with a Kolmogorov–Smirnov test, a Q-Q plot, and Levene’s test.

Categorical variables were expressed as n (%). Continuous normally distributed variables were represented by their mean and standard deviation, and continuous, non-normally distributed data by their median and interquartile range for skewed distributions. To compare categorical variables among different groups, we used Pearson’s Chi-square test or Fisher’s exact test, when appropriate. If continuous variables were normally distributed, *t*-test or ANOVA was used to compare them between two or more independent samples, respectively. Mann–Whitney U and Kruskal–Wallis tests were used if they were skewed. Predictors were evaluated with univariate and multivariable logistic regression analyses. The unique contributions of independent predictors were quantified by estimating proportional adjusted ORs. We quantified the impact of obesity on the serological response via an ordinal logistic regression model that included age, sex, comorbidities, previous infection, type of vaccine, and obesity. An ordinal logistic regression model was also used for group B (n = 126) and group C (n = 77), for quantification of the impact of bariatric surgery on the serological response. All independent variables with more than ten events and showing *p* values < 0.1 were analyzed for multivariable logistic regression analysis, by using backward elimination. The optimal prediction model was evaluated with -2Log likelihood. The significance level for baseline variables and multivariable regression analysis was set at *p*-value < 0.05. Statistical analyses were performed using IBM SPSS Statistics (IBM Corp. Released 2020. IBM SPSS Statistics for Windows, version 27.0. Armonk, NY, USA: IBM Corp) and R (Version 4.0.4) packages [17–19].

#### Data capture

The analysis was performed on a blinded data set after medical/scientific review was completed and all protocol violations had been identified, and the data set was declared complete. All data were collected in a data management system (Castor EDC, Amsterdam, The Netherlands; https://www.castoredc.com) and handled according to Good Clinical Practice guidelines, Data Protection Directive certification, and compliance with Title 21 CFR Part 11. Furthermore, the data center where all the research data are stored is ISO27001, ISO9001 certified, and Dutch NEN7510 certified.

## Results

### Baseline characteristics

In total, 276 participants were prospectively recruited from the hospital database system; n = 73 in group A (controls), n = 126 in group B (no bariatric surgery yet), and n = 77 in group C (after bariatric surgery). Mean age was 42.0 ± 14.5 years in group A, 37.3 ± 9.9 years in group B, and 39.0 ± 9.0 years old in group C. Male patients comprised 62% in group A, 29% in group B, and 35% in group C.

The mean BMI (kg $$/{m}^{2})$$ was 25.5 ± 2.2 in group A, 44.2 ± 8.6 in group B, and 31.1 ± 6.9 in group C. The BMI distribution (healthy weight, overweight or obese) varied among the groups.

Group A had no individuals with obesity. Group B consisted solely of patients with obesity (100%). Group C had patients with overweight (52%) and obesity (42%). In group C (after bariatric surgery), most patients had undergone sleeve gastrectomy (84%).

In group C, a mean ± sd reduction in BMI of 16.2 ± 8.9 kg $/{m}^{2}$ was achieved. The mean Excess Weight Loss percentage (EWL%) was 63% ± 20%, and the weight loss percentage (WL%) was 32.9 ± 12%. The mean time since bariatric surgery was 27.3 ± 17.2 months.

#### Comorbidities

At least one comorbidity was reported by 44% of the patients. The most common were hypertension (55%), diabetes mellitus (40%), and dyslipidemia (29%). The frequencies of comorbidities, dyslipidemia, and sleep apnea were significantly higher in group B than in groups A and C (Table 1).

**Table 1:** Baseline and clinical characteristics of study participants

	Control		Pre-operative		Post-operative		*p*
Characteristic	*(n* = *73)*		*(n* = *126)*		*(n* = *77)*		
Male	45_a_	62%	36_b_	29%	27_b_	35%	(< .001)
Age in years, *M* ± SD	42.0_a_ ± 14.5		37.3_b_ ± 9.9		37.0_b_ ± 9.0		(.007)
Obesity							
*BMI in kg/m*^*2*^***,**** M* ± SD	25.5_a_ ± 2.2		44.2_b_ ± 8.6		31.1_c_ ± 6.9		(< .001)
*BMI categories*							(< .001)
Healthy weight	24_a_	33%	0_b_	0%	5_c_	6%	
Overweight	49_a_	67%	0_b_	0%	40_a_	52%	
Obese	0_a_	0%	126_b_	100%	32_c_	42%	
Comorbidities							
*Any comorbidity*	24_a_	33%	66_b_	52%	32_a b_	42%	(.024)
*Multiple comorbidities*	13_a_	54%	29_a_	44%	14_a_	44%	(.663)
*Type of comorbidity*							
HTN	16_a_	22%	27_a_	21%	24_a_	31%	(.251)
DM	10_a_	14%	21_a_	17%	18_a_	23%	(.274)
Dyslipidemia	2_a_	3%	31_b_	25%	2_a_	3%	(< .001)
Sleep apnea	0_a_	0%	18_b_	14%	0_a_	0%	(< .001)
Asthma	1_a_	1%	4_a_	3%	4_a_	5%	(.418)
CVD	5_a_	7%	1_b_	1%	1_a, b_	1%	(.020)
Others	6_a_	8%	0_b_	0%	0_b_	0%	(< .001)
Vaccines							
*Type of vaccine*							(< .001)
Vector	31_a_	42%	31_b_	25%	36_a_	47%	
RNA or mRNA	2_a_	3%	67_b_	53%	6_a_	8%	
Whole-virus	40_a_	55%	28_b_	22%	35_a_	45%	
*Two doses (n* = *275)*^*a*^	73_a_	100%	91_b_	72%	67_c_	87%	(< .001)
*Time since last dose (d), Mdn (IQR)*	77_a_ (68.5)		54_a_ (90.75)		48_b_ (53.5)		(.017)
Previous infection							
*Yes*	22_a_	30%	25_a_	20%	12_a_	16%	(.080)
*Time since infection (m), M* ± SD	2.7_a_ ± 4.9		1.7_a_ ± 4		1.4_a_ ± 3.9		(.170)
Adverse events							
*Yes*	22_a_	30%	37_a_	29%	24_a_	31%	(.964)
*Type of adverse event*^*b*^							(.724)
Fever (grades 1,2)	2_a_	9%	6_a_	16%	2_a_	8%	
Fatigue and myalgia (grades 1,2)	8_a_	36%	8_a_	22%	5_a_	21%	
Pain at injection site (grades 1,2)	9_a_	41%	14_a_	38%	10_a_	42%	
Abdominal pain (grades 1,2)	3_a_	14%	9_a_	24%	7_a_	29%	
Serological response (ordinal)							(< .001)
*Negative (*< *1 U/mL)*	5_a_	7%	13_a_	10%	2_a_	3%	
*Low positive (1–5 U/mL)*	3_a_	4%	22_b_	17%	3_a_	4%	
*Medium positive (*> *5–10 U/mL)*	2_a_	3%	12_a_	10%	4_a_	5%	
*Strong positive (*> *10 U/mL)*	63_a_	86%	79_b_	63%	68_a_	88%	

*HTN*, Hypertension *DM*, Diabetes mellitus *CVD*, Chronic venous disease

Others: cancer, lupus erythematosus, pernicious anemia, renal failure, rheumatoid arthritis, thyroiditis

Within a row, groups without a common subscript letter differ at a p-value of 0.05; e.g., _a_ is different from _b_ and is not different from _ab_

^a^Only one patient received the J&J vaccine. As complete vaccination was achieved using one dose with the J&J vaccine and two doses with all other vaccines, the patient who received the J&J vaccine was excluded from the analysis

^b^Grades referenced above are according to the FDA’s guidance on adverse reactions for vaccines ^(12)^

#### Vaccines

Participants who received mRNA (Moderna and Pfizer) and vector (Johnson and AstraZeneca) vaccines were grouped together, while participants who received whole inactivated virus vaccine (Sinopharm) were a separate group. Patients in groups A and C mainly received the whole-virus and vector vaccines, whereas mRNA vaccines were the most common in group B. Time since the last complete dose was 77 ± 45 days in Group A, 73.8 ± 56.1 days in Group B, and 55.1 ± 37.1 days in Group C.

#### Previous infection

The rate of previously being infected with SARS-CoV-2 was comparable across the three groups. One-fifth (n = 59) self-reported at least one previous COVID-19 infection, and four patients (1%) reported two infections.

### Vaccine serological response

During the study, 256 (92.8%) participants had a positive serological response. Most (n = 210, 82%) responses were strongly positive, and 10.9% were low positive. The strongly positive serological response was observed in 86% of group A, 63% of Group B, and 88% of group C (*p* < 0.001).

**Fig. 1:**
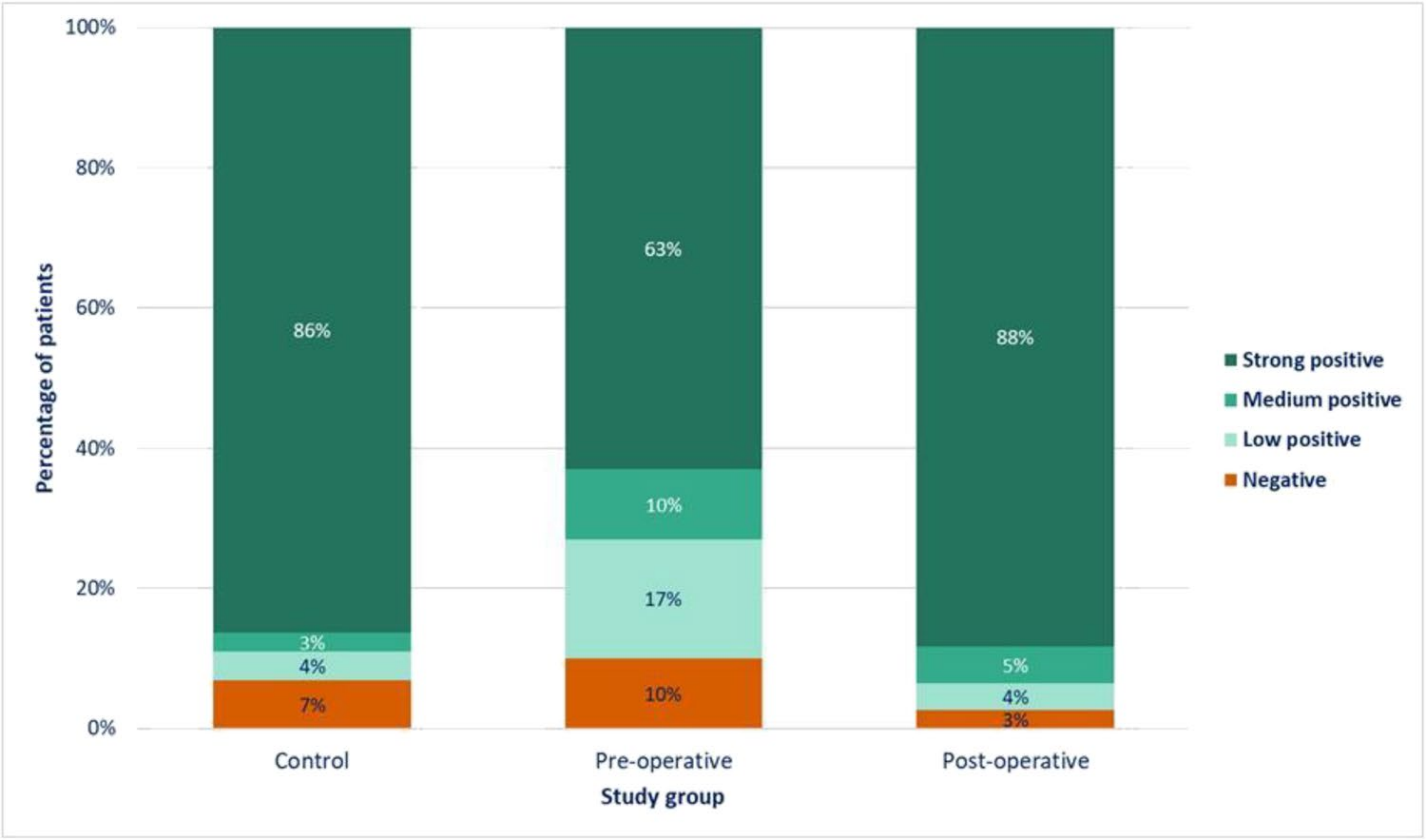
The distribution of anti-SARS-CoV-2 S protein RBD antibodies among the study groups

Figure 1 illustrates the three studied groups (control, preoperative, and postoperative) and their serological responses (four categories from negative to strong positive) against the SARS-CoV-2 S-protein RBD. Group A (control) had the highest percentage of patients with a value ≥ 250 U/mL (64%) and the highest median (interquartile range (IQR)) serological response (34.7, IQR: 3.1 to 146.9). Group B had the lowest values; 51% of patients in group B had a value ≥ 250 U/mL, and the median value in the remaining patients was 4.4 U/mL (IQR: 1.3 to 10.0). The corresponding values in group C were 58% and 6.5 U/mL (IQR: 6.5 to 71.2).

A strongly positive response was observed in 57% of patients who had received only one vaccine dose (and 80% in those who were completely vaccinated), in 63% of patients with dyslipidemia (78% in those without), and in 68% of patients with obesity (86% among patients who were healthy or underweight, and 89% among those who were overweight) (Table 2).

**Table 2:** Distribution of the serological response following COVID-19 vaccination according to general and clinical characteristics

		Vaccine serological response						Odds ratio	
	NEG		Low/med.POS		Strong POS		*Est*	*(95% CI)*	*(p-value)*
Sex									
*Female (R)*	11	7%	36	21%	121	72%			
*Male*	9	8%	10	9%	89	82%	1.70	(0.93, 3.08)	(.082)
Age in years	37.6 ± 11.5		38.9 ± 2.5		38.5 ± 11		1.00	(0.98, 1.03)	(.970)
BMI categories									
*Obese (R)*	13	8%	38	24%	107	68%			
*Healthy/underweight*	1	4%	3	11%	24	86%	2.17	(0.78, 5.99)	(.136)
*Overweight*	5	6%	5	6%	79	89%	3.55	(1.70, 7.41)	(< .001)
Comorbidities									
*No*	9	6%	24	16%	121	79%			
*Yes*	11	9%	22	18%	89	73%	1.38	(0.8, 2.39)	(.252)
No. of comorbidities									
*Single (R)*	6	9%	12	18%	48	73%			
*Multiple*	5	9%	10	18%	41	73%	1.02	(0.46, 2.26)	(.952)
DM									
*No (R)*	17	7%	40	18%	170	75%			
*Yes*	3	6%	6	12%	40	82%	1.47	(0.67, 3.21)	(.332)
Dyslipidemia									
*No (R)*	15	6%	38	16%	188	78%			
*Yes*	5	14%	8	23%	22	63%	0.46	(0.22, 0.97)	(.041)
HTN									
*No (R)*	16	8%	32	15%	161	77%			
*Yes*	4	6%	14	21%	49	73%	0.85	(0.45, 1.57)	(.598)
Type of vaccine									
*Vector (R)*	7	7%	10	10%	81	83%			
*RNA or mRNA*	4	5%	17	23%	54	72%	0.58	(0.29, 1.13)	(.109)
*Whole-virus*	9	9%	19	18%	75	73%	0.58	(0.28, 1.19)	(.138)
Complete vaccination^a^									
*Incomplete (R)*	7	16%	12	27%	25	57%			
*Complete*	13	6%	34	15%	184	80%	3.01	(1.55, 5.83)	(< .001)
Time since last dose (d)	69.3 ± 54.8		73.7 ± 45.1		68.4 ± 9.7		1	(0.99, 1)	(.601)
Previous infection									
*No (R)*	19	9%	35	16%	163	75%			
*Yes*	1	2%	11	19%	47	80%	1.39	(0.69, 2.79)	(.358)
Time since infection (m)	0.5 ± 2.1		2 ± 4.2		2 ± 4.4		1.03	(0.96, 1.11)	(.385)
Adverse events									
*No (R)*	12	6%	36	19%	145	75%			
*Yes*	8	10%	10	12%	65	78%	1.13	(0.61, 2.08)	(.703)

*NEG*, negative; *low*, low; *med*, medium; *POS*, positive; Underunder and normal-weight: 24.9 kg/m^2^, overweight: 24.9–29.99 kg/m^2^, obese:  ≥ 30.0 kg/m^2^, *Est*, estimated value; *CI*, confidence interval; *R*, reference category; *d*, days; *m*, months. ^a^Only one patient received the J&J vaccine. As complete vaccination was achieved using one dose with the J&J vaccine and two doses with all other vaccines, the patient who received the J&J vaccine was excluded from the analysis. All values are expressed as n and % or *mean* ± *SD*

As complete vaccination was achieved using one dose with the J&J vaccine and two doses with all other vaccines, cases who received the J&J vaccine (n = 1) were excluded from the analysis. The adjusted proportional odds ratios (OR) revealed that, while controlling for other factors, the odds of a stronger immune response were significantly lower in group B than in the other participants (OR 0.43, 95% CI 0.21 to 0.896), and significantly higher among those who were completely vaccinated than in those who were not (OR: 2.157, 95% CI: 1.074 to 4.334). The serological vaccine response was higher with vector or mRNA vaccines than with the whole-virus vaccine (OR = 4.37, 95% CI 1.78 to 10. 7 and OR = 2.44, 95% CI 1.09 to 5.47, respectively). The other variables in the model did not significantly impact the serological response.

### Immune response and bariatric surgery

An ordinal logistic regression model was built on Group B (n = 126) and Group C (n = 77) to quantify the impact of bariatric surgery on the immune response. As complete vaccination was achieved using one dose with the J&J vaccine and two doses with all other vaccines, cases who received the J&J vaccine (n = 1) were excluded from the analysis.

Following backward elimination, the multivariable logistic regression model included the type of vaccine, complete vaccination completeness, and duration since the last dose. Bariatric surgery increased the odds of achieving a higher serological response by a factor of 5.34 [95% CI 2.15 to 13.18] adjusted for other variables in the model. The impact on immune response did not differ by type of surgery, original BMI, or duration since surgery (Table 3).

**Table 3:** Impact of bariatric surgery on immune response following COVID-19 vaccination while adjusting for vaccine-related factors

	Adj. proportional OR^a^		
Predictors	Est	95% CI	*p*
Type of vaccine			
Vector vs. whole-virus	2.82	(1.03, 7.78)	(.045)
mRNA vs. whole-virus	2.64	(1.14, 6.11)	(.023)
Complete vaccination^b^	2.98	(1.23, 7.24)	(.016)
Time since last dose (days)	1.00	(1.00, 1.01)	(.221)
Bariatric surgery	5.34	(2.19, 13.01)	(< .001)

^a^Adjusted proportional odds ratio estimated by using multiple ordinal logistic regression to determine the independent predictors of the serological response. The estimated value (Est.) and 95% confidence intervals (CIs) are provided. ^b^Only one patient received the J&J vaccine. As complete vaccination was achieved using one dose with the J&J vaccine and two doses with all other vaccines, the patient who received the J&J vaccine was excluded from the analysis

### Adverse events

**Fig. 2:**
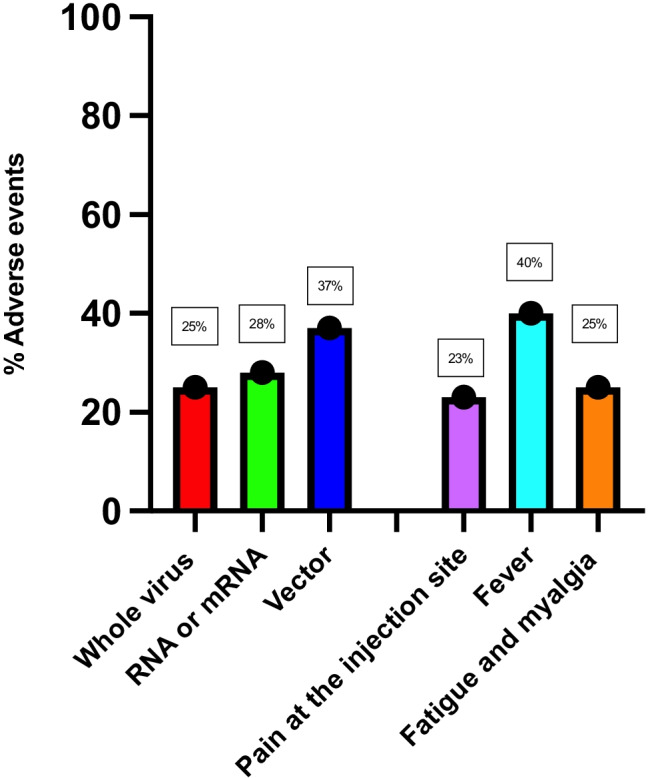
Incidence of adverse events according to vaccination type and adverse event type

Adverse events occurred in 25%, 28%, and 37% of patients who received the whole virus vaccine, RNA or mRNA, and vector vaccines, respectively. The rate of adverse events after vaccination was comparable across the three groups. Among vaccinated individuals, 83 (30%) reported one or more adverse effects, among whom 19 (23%) reported an adverse local impact (pain at the injection site). The most common systemic side-effects were fever (40%), followed by fatigue and myalgia (25%) (Fig. 2).

## Discussion

The goal of this study was to test the effectiveness of the human serological response following SARS-CoV-2 vaccination in patients who have undergone bariatric surgery (group C), and to ascertain the impact of patient characteristics on the serological response in comparison with pre-operative (group B) and control (group A) groups. The results demonstrated that the serological response increased by an adjusted OR of 5.34 after being compared to before bariatric surgery.

Previous studies have demonstrated that obesity is an independent risk factor for increased severity of complications following various viral infections, including SARS-CoV-2 infection. That finding was attributed to either an altered immune response or obesity-related comorbidities [20].

We utilized a chemiluminescent serological assay for the quantitative determination of high-affinity antibodies directed against the receptor-binding domain (RBD) on the surface spike S1 subunit of SARS‑CoV‑2. These antibodies are of particular importance because they inhibit the binding of the RBD of the S protein to the human angiotensin-converting enzyme 2 (ACE2) receptor and therefore have a strong neutralizing capacity representing real protective immunity. Numerous COVID‑19 vaccines are designed to elicit an immune response to the RBD [21].

### Comorbidities and bariatric surgery

In our study, patients with obesity were significantly less responsive (68%) than patients without obesity (86%) (*p* =  < 0.01). This is consistent with the previously reported negative correlation between obesity and the serological response, as vaccination generated a lower level of neutralizing antibodies in participants with obesity compared to those who were healthy, underweight, and overweight participants [6].

These results are also concordant with those of previous studies that indicated a poor response to hepatitis B and influenza vaccines in individuals with a higher BMI, which highlighted that those vaccines may not provide adequate protection to that population group [22]. Theories regarding the impact of obesity on the immune response to different vaccines include constant low-grade inflammation that can weaken the immune response to vaccination [22, 23].

Moreover, a low immune response may be due to obesity-associated comorbidities, such as dyslipidemia, which reportedly affects lymphocyte subsets and dendritic cells, leading to immune dysfunction. [24] In the June 2021 position statement by The Obesity Society, available clinical evidence from extensive, multicenter, global, randomized controlled trial studies on the three FDA-approved COVID-19 vaccines (Pfizer, Moderna, and Johnson & Johnson/Janssen) suggested that vaccine efficacy outcomes were not clinically different in individuals with obesity compared with individuals without obesity. However, as they stated that no formal statistical significance testing of the differences in efficacy was performed, the clinical significance of obesity on vaccine efficacy remains uncertain [25]. The potentially negative effect of obesity on the immune system and vaccine effectiveness increases the need for effective weight loss therapies. It highlights the need for continuous monitoring of the strength of the elicited immune response in this group and assessing their need for booster doses of the vaccine [26]. Bariatric surgery is a convenient and effective way to lose weight. Bariatric patients regularly voice their concerns about whether they should be vaccinated or not. Many studies proved their beneficial effects in boosting the serological response to vaccination [22, 27]. This study also contributed to this discussion, with a 68% vs. 86% efficiency on the human immune response after vaccination and an adjusted OR of 5.33 higher for bariatric surgery. Hence, bariatric surgery may increase the effect of SARS-CoV-2 vaccines in patients with obesity.

### Type of vaccine

In our study, a significantly stronger serological response was observed among patients who had received vector or mRNA vaccines rather than whole-virus vaccines (OR = 4.37, 95% CI 1.78 to 10. 7 and OR = 2.44, 95% CI 1.09 to 5.47, respectively). These findings were similar to those by the Institute of Health Metric and Evaluation (IHME), which found that efficacy in preventing disease was 94% for the Pfizer/BioNTech and Moderna (mRNA vaccines), 90% for the AstraZeneca vector vaccine, and 73% for the Sinopharm whole virus vaccine [28].

### Adverse events

In a recent systematic review of 11 trials on adverse reactions to various vaccines, most reactions were mild to moderate. Common adverse events were pain at the injection site, fever, myalgia, fatigue, and headache. Severe reactions were evident in only four trials [3]. Our study yielded similar results, with all adverse events being mild and mainly caused by vector vaccines.

### Limitations

Although our study highlights the serological response of patients post-bariatric surgery, it had certain limitations. First, we used neutralizing antibodies as a proxy for vaccine-induced protection. Although neutralizing antibodies are likely to be important in vaccine-induced protection, precise correlates of immunity are incompletely determined, and recent evidence points to a role for T cells. However, neutralizing antibodies are often much more accessible to measure than cellular responses. In addition, sufficient evidence has connected neutralizing antibody responses to SARS-CoV-2 with vaccine efficacy [29, 30]. Another possible limitation is the prospective patient selection. This may have caused some selective selection bias and thus may have influenced the results. Even though the patient selection was randomly approached, it is possible that only those with the most benefit agreed to participate. Additionally, in the possible selective selection bias, the characteristics between the group’s control vs patients with obesity/after surgery. We tried to minimize the effects of this limitation by correcting for confounding through multivariable regression analysis. In addition, the sample size of each group was small. Given that the study took place in a geographically dispersed country with approximately 100 million inhabitants, this may have influenced the outcome. However, given the level of evidence and the effect of vaccination on these groups, we can conclude that it looks like an imprecise determination of the effect size (wide confidence interval) other than underestimation of the effect. Groups of larger sample sizes might precisely determine the magnitude of the impact.

Furthermore, different types of vaccines were included without focusing on one type. Additionally, a longer follow-up is needed to study the clinical outcomes of the vaccinated individuals and whether some might develop infection later on. It is also worth mentioning that we had a maximum cutoff value of 250 U/mL of the anti-SARS-CoV-2 S protein RBD concentration measurement because estimating the titer higher than 250 U/mL would negatively impact our sample size, and consequently, the generalizability of our results. Nevertheless, enough impact on the serological response is described in the literature as a strong positive effect if the titer is higher than 10 U/mL. Also, the titers from 250 U/mL are on average measured in the literature as high responses and therefore enough for a strong response as an outcome. So, that would give enough evidence that we found the natural effect of a strong reaction on vaccination and therefore measuring higher levels above 250U/mL would not have an extra impact on the outcome [15, 31, 32].

In this study, the self-reported nature of the data might have caused inaccuracies and bias. We tried to minimize this bias by relying on vaccination records as the source for vaccination status. The history of previous infections was based on the modified WHO surveillance case definition, where a positive history was reported if the person had specific symptoms. Without laboratory confirmation, false positives were a possibility as other pathogens can cause similar respiratory illness syndromes, e.g., other coronaviruses, influenza, even during periods of the high incidence of COVID-19 disease. False negatives were also possible as most SARS-CoV-2 infections are either asymptomatic or result in mild disease [33].

## Conclusion

Vaccination with various types of COVID-19 vaccine elicited an excellent serological response among people with different BMIs with mild to moderate adverse events. Patients with lower BMI responded well to the vaccine compared to patients with obesity. The serological response increased by a factor of 5.34 following bariatric surgery, highlighting its beneficial effects. However, further follow-up is needed to monitor for later infections in patients who underwent bariatric surgery and have been vaccinated.
